# A Randomized Feasibility Study of Virtual and Face-to-Face Care Using a Novel Mobile Health Solution for Outpatient Pediatric Burns

**DOI:** 10.1093/jbcr/iraf148

**Published:** 2025-07-29

**Authors:** Allison B Frederick, Yulia Gavrilova, Natalie Koren, Courtney Tresslar, Martina Mueller, Lucas McDuffie, Steven A Kahn, Rohit Mittal, Aaron P Lesher

**Affiliations:** Department of Surgery, Medical University of South Carolina, Charleston, SC 29425, United States; Department of Surgery, Medical University of South Carolina, Charleston, SC 29425, United States; Department of Psychiatry and Behavioral Sciences, Medical University of South Carolina, Charleston, SC 29425, United States; Department of Surgery, Medical University of South Carolina, Charleston, SC 29425, United States; Department of Surgery, Medical University of South Carolina, Charleston, SC 29425, United States; College of Nursing, Medical University of South Carolina, Charleston, SC 29425, United States; Department of Public Health Sciences, Medical University of South Carolina, Charleston, SC 29425, United States; Department of Surgery, Oregon Health & Science University, Portland, OR 97239, United States; Department of Surgery, Medical University of South Carolina, Charleston, SC 29425, United States; Department of Surgery, Medical University of South Carolina, Charleston, SC 29425, United States; Department of Surgery, Medical University of South Carolina, Charleston, SC 29425, United States

**Keywords:** feasibility study, pediatric burns, outpatient burn care, telemedicine, mobile health

## Abstract

Burn injury in childhood is common, and patients often travel long distances to burn centers due to regionalization of care. To address these geographic disparities, we created a smartphone app, telemedicine optimized burn intervention (TOBI), to provide access to burn care in the outpatient setting. We conducted a randomized controlled trial to assess the feasibility, acceptability, trial methodology, and variability estimates for a larger efficacy study. Eligible pediatric burn patients were recruited in the emergency or clinic setting and randomly assigned to virtual outpatient care with TOBI or routine face-to-face (FTF) care in the clinic. Participation, study retention, compliance with prescribed burn care regimen, and clinical outcomes were assessed at baseline and each visit. After approaching 85 families, 65 (78%) patient/caregiver dyads were randomized to TOBI (*n* = 32) or FTF care (*n* = 33). The average age was 7.8 (5.4) vs 5.5 (4.6) years with 2.4 (2.1) vs 2.2 (1.6) % TBSA partial thickness burns in the TOBI and FTF groups, respectively. Treatment adherence was similar (TOBI 85% vs FTF 77%, 95% CI [−9.4, 25.7]), as were burn care completion rates (TOBI 91% vs FTF 76%, 95% CI [−0.03, 0.3]). Telemedicine optimized burn intervention patients had less pain (0.3 vs 1.0, 95% CI [−1.3, −0.1]), fewer in-person clinic visits (1.1 vs 2.1, 95% CI [−1.2, −0.7]), and shorter travel time (2.3 vs 4.0 h, 95% CI [−3.5, −0.1]). Virtual outpatient care was well-received by pediatric burn patients and caregivers, with strong engagement. The study established clinical efficacy endpoints for a future large-scale study. Clinical Trial Registration ID: 005019144.

## Graphical Abstract

**Graphical Abstract:**
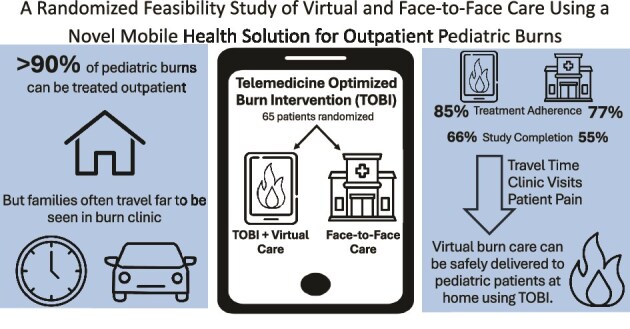

## INTRODUCTION

Pediatric burn injury remains a major public health problem, with approximately 1 million burns occurring each year in the United States, 120 000 of which require emergency medical care.^1^^,^^2^ Specialized burn care, similar to medical services delivered at verified trauma centers, has been associated with improved survival, cosmetic and functional outcome, decreased hospital costs and shorter lengths of hospital stay.^3^^,^^4^ Unfortunately, the number of American Burn Association-verified and recognized burn centers has decreased significantly over the past 30 years due to regionalization of care.^5^ The majority of the American population now lives more than 2 h by ground transportation to a verified burn center.^5^^,^^6^ With over 90% of pediatric burn patients being treated in the outpatient setting, the majority of care occurs in emergency departments and outpatient burn or plastic surgery clinics, often requiring multiple trips to the burn center to ensure that the burn heals without complication.^7^^,^^8^

**Figure 1:**
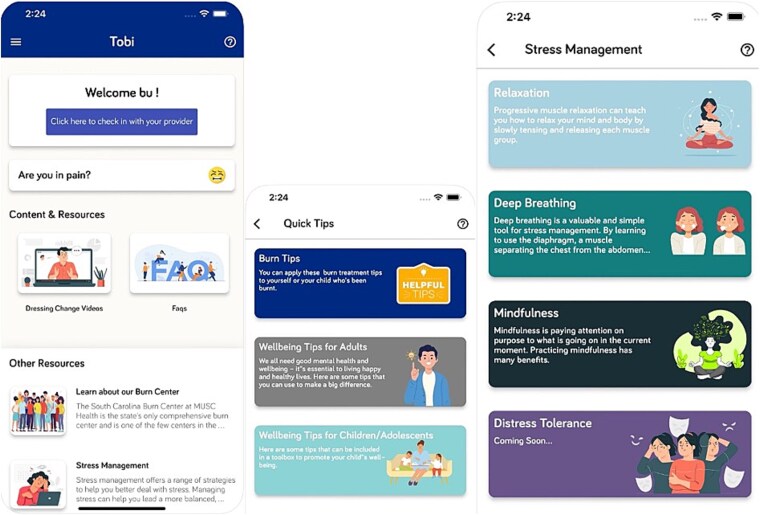
Screen Captures of the TOBI App Including Content, Resources, Tips, Frequently Asked Questions, and Well-Being/Stress Management Techniques for Patients and Caregivers

To address these geographic disparities, telemedicine optimized burn intervention (TOBI) was developed as a solution to provide expert burn care for outpatient burns remotely in the home during the acute burn phase.^9^^,^^10^ The app was designed to provide burn wound care directly through asynchronous image sharing and text messaging. This service was developed to directly address poor access to expert burn care, including high-cost burden and time commitment (eg, geographic limitations, transportation to burn centers, parking, lodging, meals and time away from school and work), particularly for patients and families in rural and medically underserved communities. Additionally, the app provides resources for patients and families, including frequently asked questions and burn wound care instructional videos (Figure 1).

Telemedicine optimized burn intervention, formerly called “Teleburn,” was initially studied in an open pilot trial (*n* = 32) conducted to examine the impact of using asynchronous communication between burn experts and the burn-injured patients’ family while the patient and caregiver are home.^9^ During the pilot study, there was a high level of acceptance by patients using it as their primary source of burn care, faster times to documented wound healing and fewer face-to-face (FTF) encounters, with higher adherence to therapy as compared to historical controls. A second stage of development was then pursued through a mixed-methods optimization study aimed at the user experience for burn providers and parent caregivers.^10^ This study found high parental and provider acceptance and usability. User feedback was then used to optimize the app and enhance user experience.

Ultimately, a rigorous scientific study is needed to validate this novel clinical care pathway as mobile health (mHealth) technology serves to shift the fundamental structure of the provider-patient relationship away from in-person care. Prior to a large-scale multicenter efficacy trial, we elected to perform a feasibility study to debug protocol methodology. The trial was designed to help develop interventions and outcome measures while identifying and addressing barriers to recruitment, retention, and procedures essential for a successful large-scale, multicenter randomized controlled trial (RCT). This is consistent with expert recommendations to test the feasibility of conducting an RCT while avoiding hypothesis-testing and yielding data to debug the methodology and assess optimal strategies for executing the RCT.^11-13^ We hypothesized that TOBI could be feasibly studied in a rigorous, prospective fashion to examine the quality of virtual burn care delivered through a novel mHealth platform.

## METHODS

### Study design

The feasibility RCT was conducted at a single quaternary care academic medical center with a pediatric burn center. The study was conducted and reported following the Consolidated Standards of Reporting Trials (CONSORT) guidelines, associated checklist uploaded as Supplementary Data 1, and the protocol for study design is registered at ClinicalTrials.gov (ID NCT 005019144). All methods were performed in accordance with relevant guidelines, and the study was approved by the Institutional Review Board (IRB) prior to starting any study procedures. Blinded randomization (1:1) was stratified by burn attending on service during the initial clinical presentation, and the randomization allocation sequence was generated and managed using REDCap electronic data capture. To avoid treatment contamination (eg, surgeons asking patients without TOBI to send them pictures of the wounds), surgeons were stratified with 32 dyads per arm, 32 study participants per burn surgeon. To minimize the likelihood that the blind was broken (ie, the next treatment assignment could be guessed), the block size was varied. Because the burn therapists and surgeons are providers, blinding at the level of provider was deemed impossible. Blinding occurred at the level of data analysis, as described below. Participants were enrolled by a designated clinical research coordinator (CRC) after identification for inclusion in the trial by the burn attending physician at the initial burn evaluation visit, either in the pediatric burn clinic or the emergency department (ED).

After obtaining informed consent, the CRC assisted parents in completing the initial questionnaires assessing attitudes toward telemedicine, healthcare access, and exposure to mHealth technology. The randomization scheme yielded at least 16 patients per treatment group for each burn provider to balance out confounding effects. Patient/caregiver dyads were followed until wounds were healed and were contacted 1-month later for poststudy surveys, including satisfaction with care and psychological recovery; psychological survey data will be published in a separate report as a secondary analysis. Participants were compensated with gift cards after the initial visit and at completion of 1-month follow-up.

### Inclusion criteria

**Figure 2:**
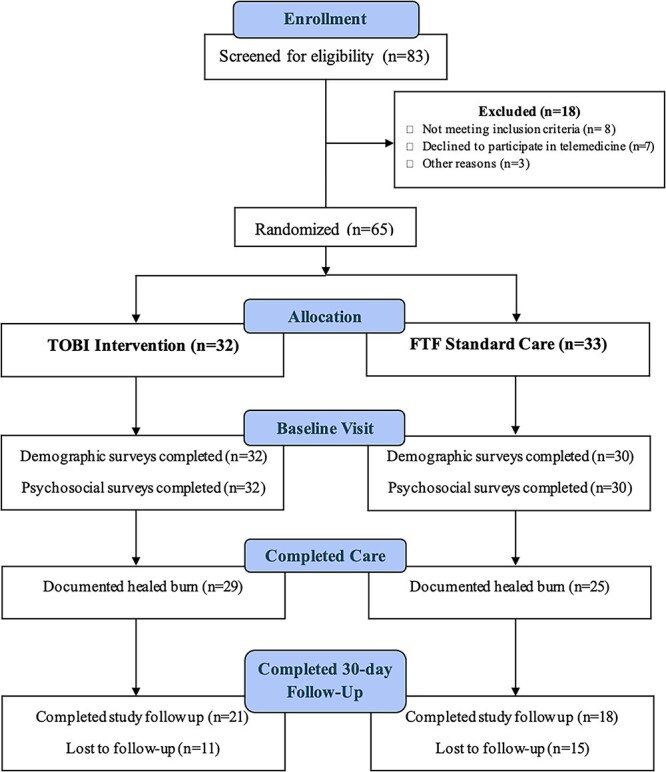
CONSORT Flow Diagram for Study Inclusion and Completion

Eligible burn-injured children were identified in the ED and pediatric outpatient burn clinic after initial in-person burn injury evaluation and were approached by members of the study team. Inclusion criteria required that: (1) patients were <18 years of age; (2) patients were diagnosed with a non-electrical, non-chemical, partial-thickness burn between 1% and 10% TBSA by a pediatric burn surgeon; (3) burns were being treated with advanced burn dressing therapy (eg, Silvadene, Mepitel); (4) burn evaluation was completed by burn surgeon within 48 hours of injury; (5) patients/caregivers were able to speak, hear, and understand English; (6) caregivers were capable of using a smart device; and (7) patients/caregivers were able to comply with outpatient clinic visits. A flow diagram for study inclusion is shown in Figure 2.

### Intervention group—TOBI

Participants assigned to TOBI were given instructions on how to download and use TOBI on a smartphone and instructed on how to interface with burn clinicians using the features of the app. In accordance with the IRB requirement to ensure patients were synchronously evaluated by a provider weekly, participants had scheduled virtual video visits with the care provider in addition to the continuous use of TOBI until the burn was healed. They were able to use other features of the app, such as messaging and sending photos of the burn and watching instructional videos as needed. Additionally, they were given instructions on how to contact the burn team through the hospital paging operator if TOBI was unavailable or if there was an emergency.

### Standard care group—FTF

Patients randomized to FTF underwent standard burn care, including a return follow-up in the burn clinic on a routine basis (usually weekly or biweekly) as prescribed by the clinical burn team and did not have access to the TOBI app or virtual visits.

### Outcome measures

We agree with Kraemer et al. that pilot feasibility studies are deficient in estimating effect size with sufficient accuracy for future study design.^14^ To this end, we first evaluated the availability of subjects (eligibility and willingness to participate) and assessed proportions approached and consented, as well as the feasibility of TOBI by evaluating adherence and retention rates. This study also measured protocol adherence and patient satisfaction at 1 month. Secondarily, measures of variability of outcomes relevant for a future RCT were obtained. Demographic information and clinical outcomes (%TBSA, time to healing, incidence of infection, change in therapy, adherence to prescribed care, and unexpected return to clinic/ED) were assessed during the baseline visit and at every subsequent weekly virtual or FTF visit until completion of wound healing. At each visit (virtual or in person), a case report form was completed by clinical providers and entered into REDCap by the CRC. At the end of the study, feasibility measures were evaluated, including participation and attrition, treatment adherence, safety, and problems associated with equipment technology and recruitment efforts. Primary study endpoints included adherence with prescribed wound care therapy, time to wound healing, total visits, time and cost savings, and time to treatment of wound complication (eg, infection, pain, non-healing) if applicable. Secondary study endpoints included pre- and poststudy validated self-report measures of patient/caregiver Attitudes Toward Technology,^15-17^ Barriers to Care,^18^ Wong-Baker Faces Pain Rating Scales^19^ and overall satisfaction with care provided using the Client Satisfaction Questionnaire-8 (CSQ-8).^20^ Additionally, app metrics, such as the number of videoconferences, messages, and photos were recorded for the TOBI group to evaluate utilization. At the completion of the study, those using TOBI also completed the psychometrically validated mHealth App Usability Questionnaire (MAUQ),^21^ which evaluates app usability, organization, interface, and functionality on a 1-7 Likert scale.

### Sample size calculation

Consistent with the intent of a feasibility RCT, the sample size for this study was determined for pragmatic reasons rather than through formal power calculation. Following the recommendations by Kraemer et al.^14^ as well as the CONSORT 2010 statement extension to randomized pilot and feasibility trials,^22^ the purpose of feasibility/pilot studies is to test recruitment processes, feasibility of the intervention and measurement as well as training protocols, data collection, and data quality control, storage, and retrieval processes.

### Statistical analysis

The primary goal of this RCT was to assess the feasibility of the proposed methodology to design a future hybrid effectiveness-implementation trial. For continuous feasibility measures (eg, number of technology help requests, patient satisfaction scores from patient surveys, etc.), frequency distributions and mean responses with standard deviation were obtained. For feasibility and outcome measures, 95% CIs were used to compare the groups. Baseline categorical demographic and clinical burn injury characteristics were summarized with descriptive statistics (frequencies, means, and SD). Data were analyzed in aggregate and in blinded fashion at the completion of the study to prevent bias. All data were analyzed using IBM SPSS Statistics (Version 29).^23^

## RESULTS

### Study population

This single-center RCT comparing TOBI (*n* = 32) with standard outpatient FTF care (*n* = 33) aimed to evaluate the feasibility of study methodology for outpatient management of partial-thickness pediatric burn injuries between January 2022 and January 2024. Evaluating the 2 cohorts, participant age was slightly older for the TOBI group (mean ± SD, range: 7.8 ± 5.4, 1-16 vs 5.5 ± 4.6, 1-16 years), with similar gender distribution, 50% vs 55% males. Percent TBSA was similar in size between the groups ([FTF vs TOBI] 2.4 ± 2.1, 1-7.5 vs 2.2 ± 1.6, 1-6). Focusing on caregiver characteristics between TOBI and FTF, most were female (mother or grandmother), in their 30s (35 vs 34 years), white (56% vs 46%), and of similar educational attainment, employment status, and income levels. On average, 78% and 70% of patient/caregiver dyads lived less than 60 miles from the study burn center, and only about 18% of families in each group lived in rural communities. In response to the Barriers to Care Questionnaire, the average reported baseline scores were similar and only noted to be a small problem (TOBI 72.6 vs FTF 64.5, 95% CI [-3.7, 19.7]); the lowest scores (denoting greater barriers to care) included issues with appointment scheduling and time waiting at the office. Additional demographic characteristics can be found in Table 1.

**Table 1:** Demographic Characteristics of Participant Dyads

**Characteristics**	**TOBI** ***n* = 32**	**FTF** ***n* = 33**
Caregiver age, y	37.4 ± 8.1	33.8 ± 7.2
Caregiver sex		
Female	32 (100%)	25 (75.8%)
Male	0 (0%)	5 (15.2)
Unknown	0 (0%)	3 (3%)
Caregiver relationship		
Mother	31 (96.9%)	24 (72.7%)
Father	0 (0%)	5 (15.2%)
Other	1 (3.1%)	1 (3%)
Caregiver race		
White	18 (56.3%)	15 (45.5%)
Black	13 (40.6%)	13 (39.4%)
Multiple/Other	1 (3.1%)	2 (6%)
Caregiver marital status		
Married	14 (43.8%)	15 (45.5%)
Single, never married	11 (34.4%)	10 (30.3%)
Other	7 (21.9%)	5 (15.2%)
Unknown	0 (0%)	3 (9.1%)
Caregiver education		
Less than High School	1 (3.1%)	1 (3%)
High School or GED	13 (40.6%)	18 (54.6%)
Associate’s degree	7 (21.9%)	3 (9.1%)
Bachelor’s degree	7 (21.9%)	4 (12.1%)
Master’s degree	4 (12.5%)	4 (12.1%)
Unknown	0 (0%)	3 (9.1%)
Caregiver employment		
Full-time job	25 (78.1%)	20 (60.6%)
Part-time job	2 (6.3%)	6 (18.2%)
Unemployed	5 (15.6%)	4 (12.1%)
Unknown	0 (0%)	3 (9.1%)
Caregiver income		
<$10 000	2 (6.3%)	3 (9.1%)
$10 000-$50 000	9 (28.2%)	14 (42.5%)
$50 000-$100 000	13 (40.6%)	10 (30.3%)
>$100 000	8 (25.0%)	3 (9.1%)
Unknown	0 (0%)	3 (9.1%)
Sole caregiver		
Yes	14 (43.8%)	20 (60.6%)
Unknown	0 (0%)	3 (9.1%)
Caregiver community		
City or urban	12 (37.5%)	16 (48.5%)
Suburban	14 (43.8%)	8 (24.2%)
Rural	6 (18.8%)	6 (18.2%)
Unknown	0 (0%)	3 (9.1%)
Caregiver distance		
<60 miles	25 (78.2%)	23 (69.7%)
>60 miles	7 (21.9%)	7 (21.3%)
Unknown	0 (0%)	3 (9.1%)
Child’s sex		
Female	15 (50%)	12 (36.4%)
Male	15 (50%)	18 (54.4%)
Unknown	0 (0%)	3 (9.1%)
Child’s age, y	7.8 ± 5.4	5.5 ± 4.6
%TBSA	2.4 ± 2.1	2.2 ± 1.6

This table compares the characteristics of caregivers and patients (dyads) in the standard and experimental groups. Continuous data are shown as mean ± 1 SD and categorical variables are shown as number (percent). Abbreviations: FTF, face-to-face; GED, general educational diploma; %TBSA, percentage total body surface area; TOBI, telemedicine optimized burn intervention; y, years.

### Feasibility metrics

**Figure 3:**
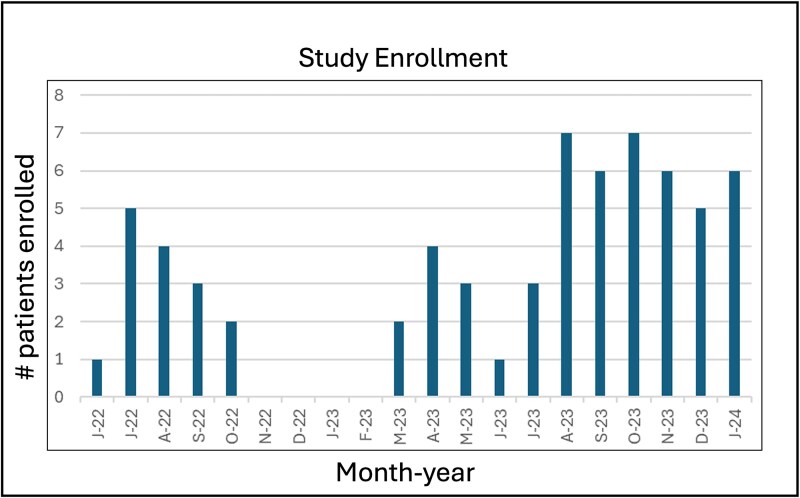
Timeline of Study Recruitment Over a 2-Year Period. Between November 2022 and February 2023, the mobile app platform was changed, and patients were not enrolled at this time

Within 2 years of study start date, a total of 65 patient/caregiver dyads were randomized to the standard FTF or experimental TOBI group and had completed the study (Figure 3). Of the 83 patients approached for study enrollment, 65 agreed and qualified to participate (78%), with 10% not qualifying for the study, 8% not interested in the study and 4% with time restraints or no phone space for app download (see Table 2). A total of 54 patients (83%) completed follow-up until burn wounds were healed (TOBI 91% vs FTF 76%, 95% CI [−0.03, 0.3]), while 39 patients (60%) completed the study (attended all appointments until healed and completed 1-month poststudy surveys) with ~10% more in the TOBI group (66% vs 55%, 95% CI [−0.1, 0.3]). The biggest reason for study attrition at the 30-day posttreatment survey time point was loss to follow-up. Importantly, no adverse events were reported, including wound infection, non-healing wound, uncontrolled pain or delay in care, and there was only 1 reported technology issue that led to a clinic appointment from the TOBI group.

### Clinical outcomes (primary and secondary endpoints)

Adherence to outpatient burn care regimen was similar in the 2 groups (TOBI 85% vs FTF 77%, 95% CI [−9.4, 25.7]), and the time to burn wound healing was nearly identical between the groups (11.4 ± 3.8 vs 11.5 ± 4.3, 95% CI [−2.4, 3.8]), as shown in Table 3. The TOBI group averaged fewer in-person visits (1.1 vs 2.1, 95% CI [−1.2, −0.7]), but the total number of clinical encounters (virtual visits + FTF visits) between the 2 groups was similar with an average of 2 visits per group. Total travel time reported by families was significantly less for the TOBI group (TOBI 2.3 ± 2.5 vs FTF 4.0 ± 3.1 h, 95% CI [−3.5, −0.1]) while time out of work and school did not significantly differ between groups (work: 1.3 ± 1.5 vs 2.0 ± 2.2, 95% CI [−1.8, 0.4]; school: 1.6 ± 1.8 vs 1.9 ± 3.0, 95% CI [−1.8, 1.2]). Additionally, reports of pain using the Wong-Baker Scale^19^ at the initial and subsequent visit by both the patient and caregiver were significantly lower in the TOBI group compared to FTF (initial visit: 1.8 vs 3.1, 95% CI [−2.6, −0.04]; subsequent visit: 0.3 vs 1.0, 95% CI [−1.3, −0.1]).

### Technology attitudes and app metrics

This study evaluated caregiver attitudes toward technology and telemedicine using a combination of validated studies^15-17^ at study enrollment and 1 month after; at baseline, both the TOBI and FTF groups scored in the mid-range as shown in Table 4 (77.0 ± 6.7 vs 76.4 ± 10.1, 95% CI [−3.7, 4.9]), and had mild improvements in attitudes toward technology after the study. Based on scores from the MAUQ on a 7-point Likert scale, caregivers reported overall high app usability scores for TOBI (6.3 ± 0.8). Evaluating the MAUQ subscales, for ease of use and satisfaction, it was rated 6.0 ± 0.16, for system arrangement it was rated 6.0 ± 0.20, and for usefulness it was rated 6.0 ± 0.23.

**Table 2:** TOBI Randomized Control Trial Feasibility Measures

Number of participants agreeing and qualifying to participate	65/83 (78%)	
Reasons for not participating (*n* = 18) Does not qualify Not interested in telemedicine Time restraints, lack of phone space	8/83 (10%) 7/83 (8%) 3/83 (4%)	
Completed burn care Total TOBI Group FTF Group	54/65 (83%) 29/32 (91%) 25/33 (76%)	
Completed 30-day follow-up Total TOBI Group FTF Group	39/65 (60%) 21/32 (66%) 18/33 (55%)	
Reason for lack of retention (*n* = 26) Lost to follow-up Not interested	**TOBI** 11/32 (34%) 0/32 (0%)	**FTF** 14/33 (42%) 1/33 (3%)
Number of participants with adverse advents	0/65 (0%)	
Number of reported technology issues	1 reported	

Abbreviations: FTF, face-to-face; TOBI, telemedicine optimized burn intervention.

**Table 3:** Primary Study Endpoints

	**TOBI** ***n* = 32**	**FTF** ***n* = 33**	**95% CI**
Treatment adherence	84.9 ± 29.1%	76.8 ± 40.1%	[−9.4, 25.7]
Time until wound healed, d	11.4 ± 3.9	11.5 ± 4.3	[−2.4, 3.8]
Wound complications	0	0	
Total Visits In-Person Visit Virtual Visit	2.2 ± 0.5, range: 1-3 1.1 ± 0.3 1.1 ± 0.6	2.1 ± 0.8, range: 1-5 2.1 ± 0.8 0	[−1.3, 0.6] [**−1.2**, **−0.7**] [**0.9, 1.3**]
Total travel time, h	2.3 ± 2.5	4.0 ± 3.1	[**−3.5**, **−0.1**]
Direct cost to caregiver^a^	$258.81 ± $390.38	$339.10 ± $568.14	[−386.86, 226.38]
Time out of work, d	1.3 ± 1.5	2.0 ± 2.2	[−1.8, 0.4]
Time out of school, d	1.6 ± 1.8	1.9 ± 3.0	[−1.8, 1.2]
Patient-reported pain scores Initial visit Subsequent visit	1.8 ± 1.7 0.3 ± 0.7	3.1 ± 3.0 1.0 ± 1.2	[**−2.6**, **−0.04**] [**−1.3**, **−0.1**]
Parent-perceived pain scores Initial visit Subsequent visit	2.2 ± 2.3 0.4 ± 0.8	2.9 ± 3.0 1.0 ± 1.4	[−2.0, 0.7] [−1.2, 0.03]

Continuous data are shown as mean ± 1 SD. Treatment adherence was defined as patient/caregiver adherence to prescribed wound care. Abbreviations: CI, confidence interval; d, days; ED, emergency department; FTF, face-to-face; h, hours; TOBI, telemedicine optimized burn intervention. Direct costs and days out of work/school were parent-reported outcomes. Bold text signifies statistically significant difference.

a Outliers were excluded if greater than 1.5 times the interquartile range.

**Table 4:** Secondary Study Endpoints

**Survey title**	**TOBI** ***n* = 32**	**FTF** ***n* = 31**	**95% CI**	**Explanation of scores**
**Barriers to Care** Baseline 1-Month Mean difference	72.6 ± 20.3 66.1 ± 21.3 −4.2 ± 22.5	64.5 ± 25.5 62.4 ± 19.8 −0.9 ± 29.2	[−3.7, 19.7] [−8.9, 16.2] [−10.6, 20.9]	Range: 0-100; higher score, fewer barriers to care
**Attitudes Toward Technology and Telemedicine** Baseline 1-Month Mean difference	77.0 ± 6.7 78.0 ± 8.4 +0.30 ± 8.9	76.4 ± 10.1 79.5 ± 6.3 +2.8 ± 11.7	[−3.7, 4.9] [−6.1, 3.1] [−3.3, 9.4]	Range: 26-130; higher score, better attitude toward technology
**MAUQ (TOBI only)**	6.3 ± 0.8			Range: 1-7; higher score, more usable app
**Client Satisfaction-8**	30.5 ± 3.0	30.8 ± 3.0	[−2.1, 1.5]	Range: 8-32; higher score, more satisfied

Survey scores are shown as mean ± 1 SD or *T*-score ± standard error as noted in the explanation column. Abbreviations: FTF, face-to-face; MAUQ, mHealth app usability questionnaire; SE, standard error; TOBI, telemedicine optimized burn intervention.

In reviewing data analytics from TOBI, the app was downloaded by all participants randomized to the TOBI group (*n* = 32) and accessed a total of 411 times during the study period. A total of 88 burn wound images were uploaded by caregivers (2.6 ± 4.6, range: 0-17), and 219 total messages (6.1 ± 8.6, range: 0-30) were sent to or from burn providers. Additionally, the frequently asked questions page was accessed 66 times (1.2 ± 3.4, range: 0-24) and the instructional burn dressing change tutorial videos were accessed a total of 38 times (0.6 ± 1.0, range: 0-10).

## DISCUSSION

This study evaluated 65 patients in a feasibility trial comparing TOBI-based virtual care to standard outpatient burn management. The methodology functioned as designed, and TOBI was delivered safely and feasibly without significant adverse events. Approximately 80% of approached families qualified and were receptive to mHealth and study participation. Of them, 83% completed follow-up to wound closure, with about 60% retained through 30-day follow-up.

While this trial was underpowered to make firm conclusions about the efficacy of TOBI, as the focus was on feasibility, several ancillary findings were identified to support the use of TOBI to broach the barriers to care in the pediatric burn population. From responses to validated questionnaires, caregivers cited challenges with scheduling convenience, taking time off work, and after-hours care—all of which TOBI intended to address. Findings from this study showed that TOBI addressed those issues with reduced average travel time for appointments by 1.7 h per family, significantly reduced the number of in-person clinic appointments from 2 to 1, and gave caregivers the ability to send messages outside of scheduled follow-up for any concerns they had. Looking further into clinical outcomes with follow-up, an unexpected finding was that caregivers and patients reported less pain during follow-up visits using TOBI compared to in-clinic visits. This finding aligns with the anxiety some children experience about doctor visits, and the comfort of home-based telemedicine likely contributed to their reduced pain.

Despite the additional access to informational videos and frequent wound evaluations with TOBI, there were no differences in the adherence to wound care or quicker wound healing times. Although no significant difference in healing times was found in the current study, both groups had an average healing time of 11 days, aligning with typical partial-thickness burn recovery.^24^ The lack of a difference in wound healing and number of clinical encounters, when compared to the difference seen in the pilot study, can likely be explained by the addition of a prescribed virtual visit with the TOBI group. To improve the safety profile of this study, the IRB required the virtual arm to include synchronous virtual visits in addition to TOBI. By adding this under the requirement of the IRB, all patients, no matter which group they were randomized to, were seen on a weekly basis until wound closure. Importantly, though, there was no delay in healing noted in either group. Additionally, this randomized study differed from the pilot data from our 2018 retrospective review of the Teleburn app, where the burn size was slightly larger on average (Teleburn 3.4 vs TOBI 2.3% TBSA).^9^ One improvement from the pilot data that was noted in this study was better adherence to burn dressing care in both the TOBI and FTF groups (TOBI: 85% vs 80%, FTF: 77% vs 64%). This was attributed to the rigorous app remodeling that occurred after our mixed-methods study, which included focus groups of caregivers and burn providers to make the app more user-friendly and helpful.^10^

Given the rise of telemedicine and mHealth over the past few years, this study stands out as one of the few RCTs focusing on improving the efficiency and efficacy of outpatient burn care. Telemedicine is feasible and has been utilized in burn care for over 40 years,^25-28^ facilitating the delivery of care to patients with burn injuries of all sizes.^29^ The rise of mHealth technology in recent years offers a promising approach to address geographic barriers to care,^30^ in addition to improving adherence to outpatient burn therapies, but there have been few validation studies.^31^

To date, no high quality, prospective RCTs have demonstrated the efficacy of telemedicine or mHealth for adult or pediatric burn patients. Additionally, a 2020 umbrella review on the effectiveness of telemedicine found it to be cost-effective across a range of adult conditions, but generalizability was limited by poor quality and reporting of studies.^32^ More high-quality studies comparing virtual and in-person care are needed, especially for this vulnerable population of children. A 2024 systemic review on virtual burn care reviewed the various time points, feasibility, and outcomes for virtual care used for consultations, discharge follow-up, and outpatient care.^33^ This review found few quality RCTs evaluating postdischarge burn care in adults focusing on quality of life and care coordination, but found that while virtual or telephone follow-up was feasible, it also showed no significant improvements in quality of life or a change in outcomes.^34^^,^^35^ While this TOBI study similarly found that ultimate outcomes and healing were not significantly different, we identified other potential markers of improved quality of life: time saved traveling, money saved on burn care, and reduced time out of work and school in the TOBI group. Ultimately, if the goal is to use mHealth and telemedicine to bridge barriers of care, the lack of outcomes differences coupled with improved quality of life markers supports the use of virtual care for pediatric burns.

While this study demonstrated the safety and feasibility of utilizing TOBI for outpatient care, several limitations or barriers to full implementation of TOBI as standard of care were encountered. Study accrual in the first year was slow with the implementation of this new technology in our clinics and ED. Additionally, there was a 4-month window where patients were not enrolled as the institution changed platforms for mHealth apps. These concerns were then quickly erased, as the remaining 50 patients were enrolled and completed the study in less than 1 year. Next, the study design was limited by the IRB’s requirement that a weekly virtual visit be used in addition to just app use of TOBI. By doing this, the IRB ensured equivalent care was received by both groups, but it also changed the primary focus from TOBI efficacy to the use of TOBI plus a virtual visit. This, in turn, reduced the overall use of TOBI compared to the higher usage in the pilot study. This finding can be addressed in further efficacy testing with an app-only arm, which is supported by the findings in this feasibility study.

Another concern that was identified and addressed during the study was the need for a burn care expert to provide timely feedback to communications sent by the caregiver, which could occur at any time of day or over the weekend. Checking for new TOBI messages was as simple as responding to a text or email on either a computer or cell phone, and many messages could be quickly addressed. Some staff even felt that responding via TOBI was easier than replying to a medical record message or calling a caregiver back. Given the ease of use, there is concern that physicians could have been biased by the type of visit, and this could not be blinded in this study, but subjective data like time to healing or identification of infection were not different between groups, while objective data like travel time and patient reported pain were not influenced by physicians conducting the virtual visit. Additionally, in this study, the risk of an emergency message being missed is low and likely equivalent, or better than in-person care, as these were small burns with no reported complications (infection and conversion to deeper burn rate of 0%). With these findings during the study, the responsibility of checking the app was transitioned from solely the burn surgeons to include trained burn nurses in the clinic. This shift has increased app usage and decreased response times.

Efficacy testing will be essential to demonstrate that remote mHealth solutions continue to evolve. The main barrier to implementation of these programs is poor coverage by third-party payors. At the start of this study, there was a seismic shift in healthcare reimbursement during the COVID-19 pandemic, designated as a public health emergency, that allowed for billing of mHealth telehealth encounters, called “virtual check-ins.” According to the Centers for Medicare & Medicaid Services, virtual check-ins are composed of brief communications with practitioners via technology on the phone or exchange of information through video or text.^36^ With the option to bill for provider time based on app usage, this argued for the use of mHealth for more efficient and rapid evaluation of burn injuries. Unfortunately, billing for virtual check-ins stopped after the emergency COVID funding ceased. At the time of publication, the use of TOBI alone was still considered a “virtual check-in” and no longer reimbursed by public and most private third-party payors. Additionally, during this RCT only English-speaking patients/caregivers were enrolled as communication via TOBI is in real time and does not currently have in-app translation or interpreters available. Unfortunately, this widens the gap in access to healthcare for already marginalized groups of patients.^37^^,^^38^ Since the end of this study, we have continued to use TOBI in our clinic and incorporated using interpreter services to offer mHealth follow-up for patients unable to return to the clinic or if they request telehealth follow-up. While this is currently an imperfect system in connecting with caregivers and their families, we hope to streamline the use of interpretive services into TOBI and mHealth-based care to bridge the access gap.

## CONCLUSION

This randomized feasibility study demonstrates the ease, safety, and utility of TOBI and telemedicine for the treatment of pediatric outpatient burn care. The study methodology resulted in an adequate level of engagement from study participants, and endpoints of clinical efficacy were established to inform the conduct of a future large-scale efficacy trial.

## Supplementary Material

completed_CONSORT_checklist_(1)_iraf148

## Notes

Author contributions: Allison B. Frederick (Data curation, Formal analysis, Investigation, Validation, Visualization, Writing—original draft, Writing—review & editing [lead]), Yulia Gavrilova (Conceptualization, Data curation, Investigation, Methodology, Project administration, Supervision, Writing—original draft, Writing—review & editing [supporting]), Natalie Koren (Data curation [supporting], Project administration [equal], Writing—review & editing [supporting]), Courtney Tresslar (Data curation [supporting], Project administration [equal], Writing—review & editing [supporting]), Martina Mueller (Formal analysis [equal], Investigation [supporting], Supervision [equal], Writing—review & editing [supporting]), Lucas McDuffie (Data curation [equal], Investigation, Project administration, Supervision, Writing—review & editing [supporting]), Steven A Kahn (Data curation, Investigation, Project administration, Supervision, Writing—review & editing [supporting]), Rohit Mittal (Data curation, Investigation, Project administration, Supervision, Writing—review & editing [supporting]), and Aaron P. Lesher (Conceptualization [lead], Data curation [equal], Funding acquisition, Investigation [lead], Methodology [equal], Project administration, Resources, Supervision [lead], Writing—original draft, Writing—review & editing [equal])

Funding: The work was funded by the Eunice Kennedy Shriver National Institute of Child Health and Human Development of the National Institutes of Health (K23 MH107641).

Conflict of interest statement: Dr. Lesher submitted a record of invention for the TOBI app, but there are no current patents issued and MUSC owns the right to commercially license TOBI, although no money has been made from the app. The authors otherwise declare no conflicts of interest.

Meeting presentation: American Pediatric Surgical Association Annual Meeting; Montreal, Canada, May 2025.
